# Development of Fast Dispersible Aceclofenac Tablets: Effect of Functionality of Superdisintegrants

**DOI:** 10.4103/0250-474X.41452

**Published:** 2008

**Authors:** C. Mallikarjuna Setty, D. V. K. Prasad, V. R. M. Gupta, B. Sa

**Affiliations:** Department of Pharmaceutics, N.E.T. Pharmacy College, Raichur-584 103, India; 1 Department of Pharmaceutical Technology, Jadavpur University, Kolkata-700 032, India

**Keywords:** Fast dispersible tablets, aceclofenac, croscarmellose sodium, sodium starch glycolate, crospovidone, disintegration time, dissolution

## Abstract

Aceclofenac, a non-steroidal antiinflammatory drug, is used for posttraumatic pain and rheumatoid arthritis. Aceclofenac fast-dispersible tablets have been prepared by direct compression method. Effect of superdisintegrants (such as, croscarmellose sodium, sodium starch glycolate and crospovidone) on wetting time, disintegration time, drug content, *in vitro* release and stability parameters has been studied. Disintegration time and dissolution parameters (t_50%_ and t_80%_) decreased with increase in the level of croscarmellose sodium. Where as, disintegration time and dissolution parameters increased with increase in the level of sodium starch glycolate in tablets. However, the disintegration time values did not reflect in the dissolution parameter values of crospovidone tablets and release was dependent on the aggregate size in the dissolution medium. Stability studies indicated that tablets containing superdisintegrants were sensitive to high humidity conditions. It is concluded that fast-dispersible aceclofenac tablets could be prepared by direct compression using superdisintegrants.

Aceclofenac, (2-[2-[2-(2,6-dichlorophenyl)aminophenyl]acetyl]oxyacetic acid), a nonsteroidal antiinflammatory drug (NSAID) has been indicated for various painful indications^1^ and proved as effective as other NSAIDs with lower indications of gastro-intestinal adverse effects and thus, resulted in a greater compliance with treatment^2^. Aceclofenac is practically insoluble. For poorly soluble orally administered drugs, the rate of absorption is often controlled by the rate of dissolution. Clear aceclofenac-loaded soft capsules have been prepared to accelerate the absorption^3^. The rate of dissolution can be increased by increasing the surface area of available drug by various methods (micronization, complexation and solid dispersion)^4^. The dissolution of a drug can also be influenced by disintegration time of the tablets. Faster disintegration of tablets delivers a fine suspension of drug particles resulting in a higher surface area and faster dissolution.

Of all the orally administered dosage forms, tablet is most preferred because of ease of administration, compactness and flexibility in manufacturing. Because of changes in various physiological functions associated with aging including difficulty in swallowing, administration of intact tablet may lead to poor patient compliance and ineffective therapy. The paediatric and geriatrics patients are of particular concern. To overcome this, dispersible tablets^5^ and fast-disintegrating tablets^6^ have been developed. Most commonly used methods to prepare these tablets are; freeze-drying/Lyophilization^7^, tablet molding^8^ and direct-compression methods^9^. Lyophilized tablets show a very porous structure, which causes quick penetration of saliva into the pores when placed in oral cavity^7^,^10^. The main disadvantages of tablets produced are, in addition to the cost intensive production process, a lack of physical resistance in standard blister packs and their limited ability to incorporate higher concentrations of active drug^5^. Moulded tablets dissolve completely and rapidly. However, lack of strength and taste masking are of great concern^8^,^11^. Main advantages of direct compression are, low manufacturing cost and high mechanical integrity of the tablets^9^,^12^. Therefore, direct-compression appears to be a better option for manufacturing of tablets. The fast disintegrating tablets prepared by direct compression method, in general, are based on the action established by superdisintegrants such as croscarmellose sodium, crospovidone and sodium starch glycolate. The effect of functionality differences of the superdisintegrants on tablet disintegration has been studied^13^. The objective of the present work was to develop fast dispersible aceclofenac tablets and to study the effect of functionality differences of superdisintegrants on the tablet properties and to provide information on the storage conditions of these tablets.

## MATERIALS AND METHODS

Aceclofenac (Aristo Pharmaceuticals Ltd, Mumbai, India), croscarmellose sodium, sodium starch glycolate, and microcrystalline cellulose (Maple Biotech Pvt Ltd., Pune, India), aspartame (Ranbaxy, New Delhi, India). Crospovidone (Concertina Pharma Pvt., Ltd, Hyderabad, India). Talc and magnesium stearate were purchased from S. D. Fine Chem Ltd., Mumbai India.

### Blending and tableting:

Tablets containing 100mg of aceclofenac were prepared by direct compression method and the various formulae used in the study are shown in Table 1. The drug, diluents, superdisintegrant and sweetener were passed through sieve # 40. All the above ingredients were properly mixed together (in a poly-bag). Talc and magnesium stearate were passed through sieve # 80, mixed, and blended with initial mixture in a poly-bag. The powder blend was compressed into tablets on a ten-station rotary punch-tableting machine (Rimek Mini Press-1) using 7 mm concave punch set.

**TABLE 1:** FORMULAE USED IN THE PREPARATION OF TABLETS

Ingredients (mg)	CC I	CC II	CC III	CC IV	CP I	CP II	CP III	CP IV	SSG I	SSG II	SSG III	SSG IV
Aceclofenac	100	100	100	100	100	100	100	100	100	100	100	100
Croscarmellose sodium	4	8	16	24	--	--	--	--	--	--	--	--
Crospovidone	--	--	--	--	4	8	16	24	--	--	--	--
Sodium starch glycolate	--	--	--	--	--	--	--	--	4	8	16	24
Micro crystalline cellulose	50	50	50	50	50	50	50	50	50	50	50	50
D-Mannitol	30	26	18	10	30	26	18	10	30	26	18	10
Aspartame	10	10	10	10	10	10	10	10	10	10	10	10
Talc	4	4	4	4	4	4	4	4	4	4	4	4
Magnesium sterate	2	2	2	2	2	2	2	2	2	2	2	2

### Evaluation of dispersible tablets:

Tablets were evaluated for weight variation, hardness, friability, thickness and disintegration time^14^, wetting time^15^ and stability^16^. In weight variation test, twenty tablets were selected at random and average weight was determined using an electronic balance (Shimadzu, AX200, Japan). Tablets were weighed individually and compared with average weight. The Pfizer hardness tester and the Roche friabilator were used to test hardness and friability loss respectively. Disintegration time was determined using USP tablet disintegration test apparatus (ED2L, Electrolab, India) using 900 ml of distilled water without disk at room temperature (30°)^13^. Thickness of tablet was determined by using dial caliper (Mitutoya, Model CD-6 CS, Japan). To measure wetting time of tablet, a piece of tissue paper was folded twice and placed in a small Petri dish containing sufficient water. A tablet was kept on the paper and the time for complete wetting of tablet was measured.

### Stability studies:

The stability of selected formulations was tested according to International Conference on Harmonization guidelines for zone III and IV. The formulations were stored at accelerated (40± 2º/75±5% RH) and long-term (30±2º/65±5% RH) test conditions in stability chambers (Lab-Care, India) for six months following open dish method^17^. At the end of three months, tablets were tested for disintegration time, hardness friability, thickness, drug content and moisture uptake.

### Dissolution Study:

*In vitro* release of aceclofenac from tablets was monitored by using 900 ml of SIF (USP phosphate buffer solution, pH 7.4) at 37±0.5° and 75 rpm using programmable dissolution tester [Paddle type, model TDT-08L, Electrolab, (USP), India]. Aliquots were withdrawn at one minute time intervals and were replenished immediately with the same volume of fresh buffer medium. Aliquots, following suitable dilutions, were assayed spectrophotometrically (UV-1700, Shimadzu, Japan) at 274 nm.

### Statistical analysis:

Each tablet formulation was prepared in duplicate, and each analysis was duplicated. Effect of formulation variables on disintegration time and release parameters (t_50%_ and t_80%_) were tested for significance by using analysis of variance (ANOVA: single factor) with the aid of Microsoft^®^ Excel 2002. Difference was considered significant when *P* < 0.05.

## RESULTS AND DISCUSSION

Since, the flow properties of the powder mixture are important for the uniformity of mass of the tablets, the flow of the powder mixture was analyzed before compression to tablets. Low Hasner`s ratio (≤1.32), compressibility index (≤24.68) and angle of repose (≤18.13) values indicated a fairly good flowability of powder mixture. As the tablet powder mixture was free flowing, tablets produced were of uniform weight with acceptable weight variation (≤4.68%) due to uniform die fill. Hardness (3.63-4.31 kg/cm^2^) and friability loss (0.15-0.72 %) indicated that tablets had a good mechanical resistance. Drug content was found to be high (≥96.2%) and uniform (coefficient of variation between 0.89-2.56%) in all the tablet formulations.

**Fig. 1:**
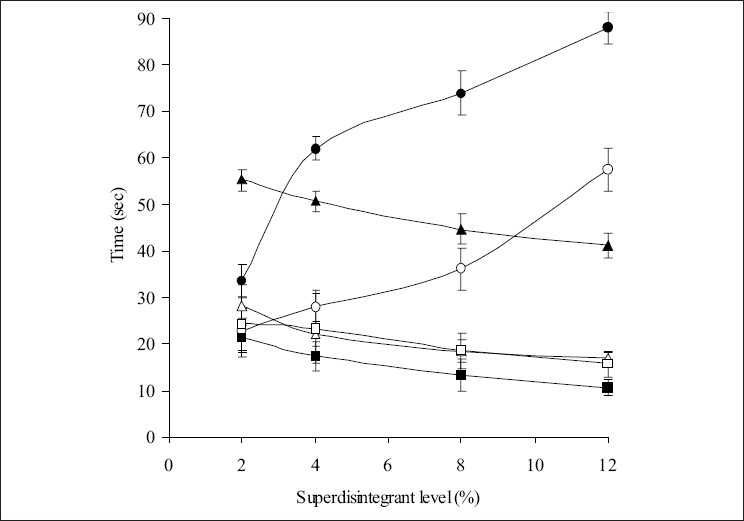
Effect of concentrations of sodium starch glycolate (—◦—), croscarmellose sodium (—Δ—) and crospovidone (—□—) on disintegration time and sodium starch glycolate (—•—), croscarmellose sodium (—▲—) and crospovidone (—■—) on wetting time of various formulations.

The most important parameter that needs to be optimized in the development of fast dispersible tablets is the disintegration time of tablets. In the present study, all the tablets disintegrated in ≤57.5 sec fulfilling the official requirements (<3 min) for dispersible tablets^18^. Fig. 1 depicts the disintegration behavior of the tablets in water. It is observed that the disintegration time of the tablets decreased (from 28.25 to 17 sec) (*P*<0.05) with increase in the level of croscarmellose sodium. In case of tablets containing crospovidone, increasing level of crospovidone had no effect (*P*>0.05) on the disintegration times of the tablets. However, disintegration time increased (*P*<0.05) with increase in the level of sodium starch glycolate in the tablets. It indicates that increase in the level of sodium starch glycolate had a negative effect on the disintegration of the tablets. At higher levels, formation of a viscous gel layer by sodium starch glycolate^19^ might have formed a thick barrier to the further penetration of the disintegration medium and hindered the disintegration or leakage of tablet contents. Thus, tablet disintegration is retarded to some extent with tablets containing sodium starch glycolate. Comparatively, disintegration times of the tablets containing crospovidone<croscarmellose sodium<sodium starch glycolate. The disintegration times of crospovidone containing tablets are comparatively lower than those containing croscarmellose sodium and sodium starch glycolate. The faster disintegration of crospovidone tablets may be attributed to its rapid capillary activity and pronounced hydration with little tendency to gel formation^20^. Thus, these results suggest that the disintegration times can be decreased by using wicking type of disintegrants (crospovidone).

Since the dissolution process of a tablet depends upon the wetting followed by disintegration of the tablet, the measurement of wetting time may be used as another confirmative test for the evaluation of dispersible tablets. Fig. 1 depicts the wetting times for tablets prepared with three superdisintegrants. Wetting times of the tablets did not change (*P*>0.05) with increase in the croscarmellose sodium from 2-4%. However, wetting times decreased (*P*<0.05) with increase in the level of croscarmellose above 4%. A significant decrease (*P*<0.05) in the wetting times is seen with increase in the level of crospovidone (4 to 12%). It is interesting to note that wetting times increased (*P*<0.05) with increase in the level of sodium starch glycolate from 2% to 12% in the tablets. Thus wetting times of tablets with crospovidone<croscarmellose sodium<sodium starch glycolate. These results are in consistent with disintegration test results.

**Fig. 2:**
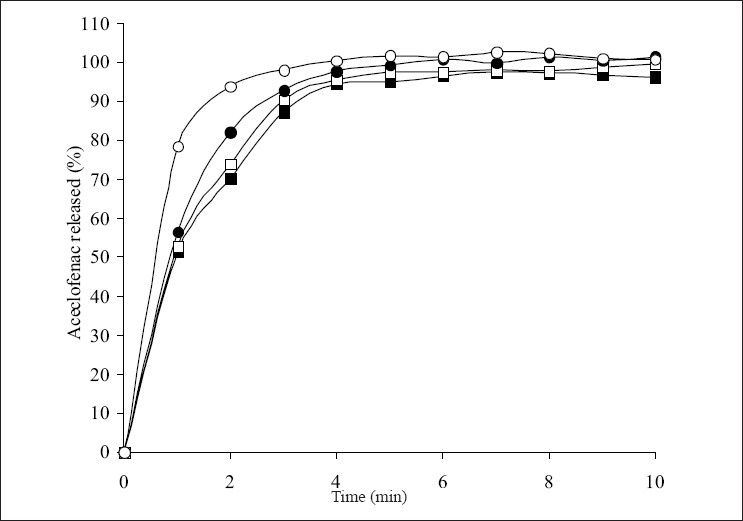
Effect of croscarmellose sodium level on the release of aceclofenac. Levels of croscarmellose sodium are 2% (—■—), 4% (—□—), 8% (—•—) and 12% (—◦—). Max ± SD = 5.3, n=3.

**Fig. 3:**
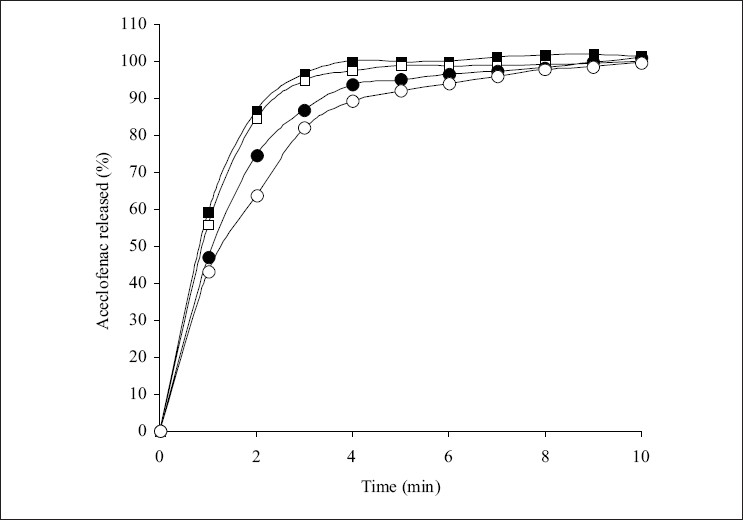
Effect of sodium starch glycolate level on the release of aceclofenac Levels of sodium starch glycolate are 2% (—■—), 4% (—□—), 8% (—•—) and 12% (—◦—). Max ± SD = 5.3, n=3.

**Fig. 4:**
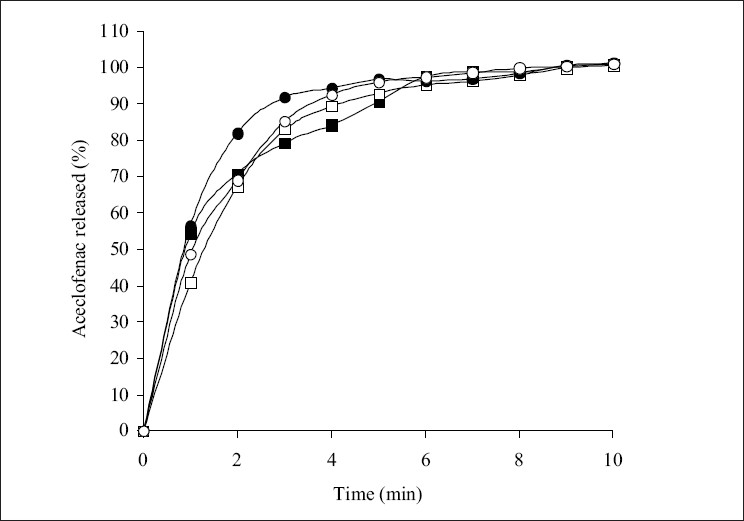
Effect of crospovidone level release of aceclofenac. Levels of crospovidone are 2% (—■—), 4% (—□—), 8% (—•—) and 12% (—◦—). Max ± SD = 5.3, n=3.

**Fig. 5:**
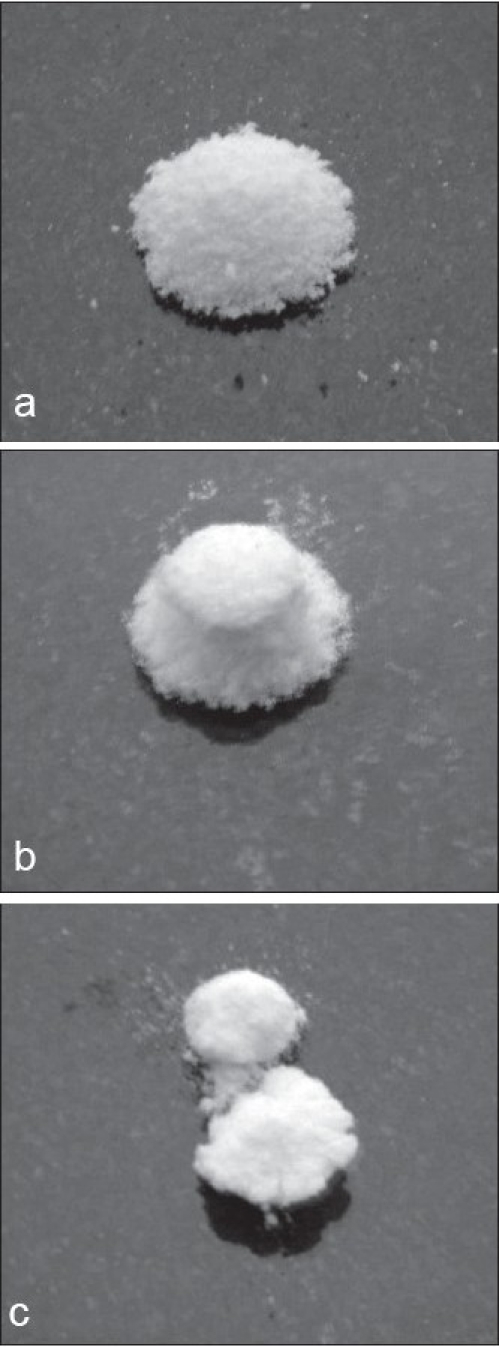
Photographs showing disintegration of tablets in water after 20 sec. Tablets were prepared with (a) croscarmellose sodium starch glycolate, (c) crospovidone.

The influence of superdisintegrants on the dissolution of aceclofenac from the tablets is shown in figs. 2-4. The t_50%_ and t_80%_ (time for 50% and 80% of release) values decreased (*P*<0.05) with increase in the level of croscarmellose sodium. However, t_50%_ and t_80%_ values increased (*P*<0.05) with increase in the level of sodium starch glycolate. While t_50%_ and t_80%_ values did not change (*P*>0.05) with increase in the level of crospovidone. These results indicated that dissolution parameter values of croscarmellose sodium and sodium starch glycolate containing tablets are in consistent with the disintegration time values observed. However, disintegration time values observed with crospovidone tablets are not predictable of dissolution of the drug. The rapid increase in dissolution of aceclofenac with the increase in croscarmellose sodium may be attributed to rapid swelling and disintegration^20^ of tablet into apparently primary particles^13^ (fig. 5a). While, tablets prepared with sodium starch glycolate, disintegrate by rapid uptake of water, followed by rapid and enormous swelling^20^ into primary particle but more slowly^13^ (fig. 5b) due to the formation of a viscous gel layer by sodium starch glycolate^19^. Crospovidone exhibits high capillary activity and pronounced hydration with a little tendency to gel formation^20^ and disintegrates the tablets rapidly but into larger masses of aggregated particles^13^ (fig. 5c). Thus, the differences in the size distribution generated and differences in surface area exposed to the dissolution medium with different superdisintegrants rather than speed of disintegration of tablets may be attributed to the differences in the t_50%_ and t_80%_ values with the same amount of superdisintegrants in the tablets. Thus, although the disintegration times were lower in crospovidone containing tablets, comparatively higher t_50%_ and t_80%_ values were observed due to larger masses of aggregates.

When tablets were kept at real time (30±2º/65±5% RH) and accelerated (40±2º/75±5% RH) storage conditions, both disintegration time and hardness values decreased significantly indicating that tablets have lost the mechanical integrity leading to more friability loss (Table 2). Increase in thickness of all tablets was noticed particularly pronounced in crospovidone tablets. These results indicate that, at higher relative humidity, tablets containing high concentration of superdisintegrants get softened and hence, must be protected from atmospheric moisture. As crospovidone tablets absorbed larger amount of moisture, tablets became fragile and developed cracks. After stability test period, some portion of the tablet edges was removed and hence, drug content, hardness, friability and disintegration tests could not be conducted on these tablets.

**TABLE 2:** STABILITY STUDY DATA OF THE TABLET FORMULATIONS

Formulation	Hardness	Friability	Thickness	Disintegration	Drug content	Increase
	(Kg/cm^2^)	(%)	(mm)	time (sec)	(%)	in weight
	(±SD), n=6	(±SD), n=10	(±SD), n=10	(±SD), n=6	(±SD), n=4	(%)
CC IV before stability study	4.5(0.42)	0.354(0.01)	4.38(0.05)	22(1.41)	99.23(2.64)	0
SSG IV before stability study	3.5(0.56)	0.476(0.04)	4.43(0.0)	68(4.65)	98.84(3.24)	0
CP IV before stability study	3.5(0.63)	0.29(0.07)	4.48(0.05)	18(2.75)	98.57(1.36)	0
CC IV 30°/						
65 % RH	4(0.41)	0.486(0.13)	4.6(0.05)	16(4.42)	97.3(2.83)	1.406
SSG IV 30°/ 65 % RH	3(0.74)	0.583(0.07)	4.6(0.05)	52(2.38)	98.76(3.24)	1.489
CP IV 30°/						
65 % RH	NA	NA	5.5(0.17)	NA	NA	2.836
CC IV 40°/ 75% RH	3.5(0.56)	0.564(0.22)	4.6(0.05)	14(4.34)	99.23(2.68)	1.617
SSG IV 40°/ 75% RH	3(0.68)	0.683(0.14)	4.9(0.17)	38(3.65)	98.84(4.32)	1.730
CP IV 40°/ 75% RH	NA	NA	6.2(0.17)	NA	NA	3.092

NA - not applicable

It is concluded that, although functionality differences existed between the superdisintegrants, the fast dispersible aceclofenac tablets could be prepared by using any of the superdisintegrants used. The dissolution parameters were consistent with disintegration times of croscarmellose sodium and sodium starch glycolate containing tablets. However, disintegration time values of crospovidone tablets were not correlating with dissolution profiles. Dispersible tablets prepared with superdisintegrants must be protected from atmospheric moisture.

## References

[1] Hinz B, Auge D, Rau T, Rietbrock S, Brune K, Werner U (2003). Simultaneous determination of aceclofenac and three of its metabolites in human plasma by high-performance liquid chromatography. Biomed Chromatogr.

[2] Legrand E (2004). Aceclofenac in the management of inflammatory pain. Exp Opin Pharmacother.

[3] Yong CS, Oh YK, Lee KH, Park SM, Park YJ, Gil YS (2005). Trials of clear aceclofenac- loaded soft capsules with accelerated oral absorption in human subjects. Int J Pharm.

[4] Martin A (1993). Physical pharmacy.

[5] Schiermeier S, Schmidt PC (2002). Fast dispersible ibuprofen tablets. Eur J Pharm Sci.

[6] Mizumoto T, Masuda Y, Yamamoto T, Yonemochi E, Tarada K (2005). Formulation design of a novel fast-disintegrating tablet. Int J Pharm.

[7] Virley P, Yarwood R (1990). Zydis-A novel fast dissolving dosage form. Manuf Chem.

[8] Dobetti L (2000). Fast-melting tablets: Developments and technologies. Pharm Technol Eur.

[9] Bi Y, Sunada H, Yonezawa Y, Danjo K, Otsuka A, Iida K (1996). Preparation and evaluation of a compressed tablet rapidly disintegrating in the oral cavity. Chem Pharm Bull.

[10] Patrick K, Sang KW (1997). Method of making freeze-dried dosage form.

[11] Chang RK, Guo X, Burnside B, Couch R (2000). Fast-dissolving tablets. Pharm Technol.

[12] Takao M, Yoshinori M, Muneo F (1996). Intrabuccally dissolving compressed mouldings and production process thereof.

[13] Zhao N, Augsburger LL (2005). Functionality comparison of 3 classes of superdisintegrants in promoting aspirin tablet disintegration and dissolution. AAPS PharmSciTech.

[14] (2004). United States Pharmacopoeia.

[15] Sunada H, Bi YX, Yonezawa Y, Danjo K (2002). Preparation, evaluation and optimization of rapidly disintegrating tablets. Powder Technol.

[16] Medwick T, Baliely LC (1998). A Procedure for conducting open-dish studies. Pharm Forum.

[17] Marais AF, Song M, de Villers MM (2003). Effect of compression force, humidity and disintegrant concentration on the disintegration and dissolution of directly compressed furosemide tablets using croscarmellose sodium as a disintegrant. Trop J Pharm Res.

[18] (2001). European department for the quality of medicines. European Pharmacopoeia.

[19] Bolhuis GK, Zuurman K, te Wierik GH (1997). Improvement of dissolution of poorly soluble drugs by solid deposition on a superdisintegrant: II, The choice of superdisintegrants and effect of granulation. Eur J Pharm Sci.

[20] Rowe RC, Sheskey PJ, Weeler PJ (2003). Handbook of pharmaceutical excipients.

